# Radiation environment in exploration-class space missions and plants’ responses relevant for cultivation in Bioregenerative Life Support Systems

**DOI:** 10.3389/fpls.2022.1001158

**Published:** 2022-09-23

**Authors:** Veronica De Micco, Carmen Arena, Luca Di Fino, Livio Narici

**Affiliations:** ^1^ Laboratory of Plant and Wood Anatomy, Department of Agricultural Sciences, University of Naples Federico II, Naples, Italy; ^2^ Laboratory of Ecology, Department of Biology, University of Naples Federico II, Naples, Italy; ^3^ Physics Department, University of Rome “Tor Vergata”, Rome, Italy

**Keywords:** cosmic radiation, functional food, ionizing radiation, life support systems, plant structure and eco-physiology, radioresistance, space exploration, space greenhouses

## Abstract

For deep space exploration, radiation effects on astronauts, and on items fundamental for life support systems, must be kept under a pre-agreed threshold to avoid detrimental outcomes. Therefore, it is fundamental to achieve a deep knowledge on the radiation spatial and temporal variability in the different mission scenarios as well as on the responses of different organisms to space-relevant radiation. In this paper, we first consider the radiation issue for space exploration from a physics point of view by giving an overview of the topics related to the spatial and temporal variability of space radiation, as well as on measurement and simulation of irradiation, then we focus on biological issues converging the attention on plants as one of the fundamental components of Bioregenerative Life Support Systems (BLSS). In fact, plants in BLSS act as regenerators of resources (i.e. oxygen production, carbon dioxide removal, water and wastes recycling) and producers of fresh food. In particular, we summarize some basic statements on plant radio-resistance deriving from recent literature and concentrate on endpoints critical for the development of Space agriculture. We finally indicate some perspective, suggesting the direction future research should follow to standardize methods and protocols for irradiation experiments moving towards studies to validate with space-relevant radiation the current knowledge. Indeed, the latter derives instead from experiments conducted with different radiation types and doses and often with not space-oriented scopes.

## Introduction

Radiation effects on the crew during space voyages must be reduced under a pre-agreed limit to allow for human deep space exploration. These effects can be direct (on the astronauts themselves) or indirect, acting on items that are indispensable for the crew life, such as Bioregenerative Life Support Systems (BLSS). In such systems, the biotic component (e.g., consumers, producers and decomposers) is integrated with physical/chemical processes to achieve a self-sustaining system allowing the regeneration of resources, fundamental to solve the issues of resupply from Earth and waste management (De Pascale et al., 2021). In BLSS, the problem of space radiation which may affect both the organisms and the electronic components of spacecrafts cannot be disregarded.

In this paper, we focus our attention on the main issues related to the radiation spatial and temporal variability as well as on the difficulty in identifying a standard behavior of plants in response to ionizing radiation due to the multiplicity of studies conducted with scopes which were not space-oriented. Indeed, most available information derives from ground-based experiments in which plants or plant parts have been exposed to photon-type radiation and not to charged particles (i.e., protons and heavy ions constituting the most of space radiation). Moreover, even when experimental irradiation has been performed with charged particles, there is still the issue of poor space fidelity of the ‘radiation analog’ accelerators. In this paper, when not specified, the reported study cases are referred to plants’ responses to photon-type radiation, but we underline the need for the scientific community to converge towards common standardization schemes in future experiments to test the effects of radiation. The validation of results obtained until now with space-relevant radiation is the sole way to finely forecast the plant behavior and to define the true cultivation requirements to obtain “well-functioning” plants efficiently providing the regeneration of resources and food production in BLSS for Space exploration.

## Space radiation and the mitigation issue

Space radiation (Durante and Cucinotta, 2011) is mostly composed by Galactic Cosmic Rays (GCR) and Solar Particle Events (SPE). The former is composed mostly by ions of all elements, from hydrogen to iron (the fluxes drop significantly for the charge Z>26). About 85% of these are protons, 14% helium ions, and 1% heavier ions. The heavier ions, however, are more effective in damaging living tissues, so their contribution to the risk is comparable to the one caused by proton flux. The spectra of all ions peaks at about 1-2 GeV/nucleon, and extend from low energies (a few MeV) well beyond the TeV region. At these two ends the fluxes are negligible, and at the low end the ions are most likely stopped by the habitat shield. GCR are isotropic: each volume is hit by radiation from all direction with equal intensity, and they depend on time because of the solar activity: they are maximum during low solar activity and minimum during high solar activity. The solar cycle extends to 11 years. The difference in dose rate due to the GCR between solar maximum and solar minimum may reach a factor of 2 (Berger et al., 2020). The radiation rate (number of hits per unit of time, unit surface) is small, many order of magnitude smaller when compared with nuclear bombs or nuclear disasters, or with the radiation rate used during hadron-therapy, even in the tails of the radiation distribution.

SPE are sporadic, of short duration (hours, days), intense (orders of magnitudes higher rates than GCR), non-foreseeable, and mostly constituted by protons with energy spectra peaking about one order of magnitude lower than GCR (Larosa et al., 2011; Semkova et al., 2013; Berrilli et al., 2014; Di Fino et al., 2014; Berger et al., 2018; Narici et al., 2018). SPEs travel through the solar system around the solar magnetic field lines, and therefore each SPE present a peculiar direction hitting specific points in the solar system. The probability of SPE occurrence is higher during periods of higher solar activity.

Thus, in sum, i) GCR are always present, isotropic, energetic, low rate; they are maximum during periods of minimum solar activity. ii) SPE are unpredictable, short duration, with directionality, lower energy and higher rates than GCR; they are less probable during periods of minimum solar activity.

When GCR and SPE radiation enter a shield, such as the outer hull of a spacecraft, they produce a flux of secondary radiation, of composition and spectra different from the primary external radiation. Among all the secondary radiation, for radiation protection issues, neutrons deserve probably the most careful consideration.

Mitigating radiation effects on humans in space is a difficult endeavor (Durante and Cucinotta, 2011). The two most important approaches often used on Earth, namely increasing distance from the source and minimizing the time of exposure, are not applicable in space.

Radiation is not decreasing with distance as with nuclear explosions or reactor spills, or in general in any situation where the source is localized. As already mentioned, the GCR are isotropic and impact with a target in any place in the solar system with similar intensity from all directions. Even the solar particle events coming from the sun do not feature very significant reductions with distance, being “channeled” by the solar magnetic field to target.

Furthermore, the time of exposure cannot be used as a minimization variable. The travel time will be reduced to a minimum anyhow (due to several concurrent and obvious advantages in doing so). The duration of the presence in space, for example on the surface of the planet to be explored, should not be reduced as the scientific value of the mission would be reduced accordingly.

In a sense, the mitigation of the radiation effects on humans should be performed in such a way to allow the human beings to live in deep space indefinitely.

The only mitigation procedures we are left with are: i) reduce the amount of radiation reaching the human body (shielding) or ii) provide means to make the human body resilient to radiation. This last approach can be further classified in two ways: making us more radiation resistant, or/and, providing means to counteract the negative effects of radiation. Pharmacological, nutritional, physiological countermeasures may eventually play these roles.

Focusing on the first mitigation procedure (shielding) we can proceed in two ways: interposing materials between the radiation and the human body (passive shielding) or deflecting the radiation in such a way it does not reach the human body. This last method (active shielding) is very promising but not practical yet (Battiston et al., 2011): technological breakthroughs are needed to build efficient radiation deflectors to be used in space travels. So, we are left with passive shielding. However, cosmic radiation is energetic, and the ions travel at speeds close to light speed. This means that a lot of radiation may well pass through the shielding material, and also that the interactions of the same radiation with the shielding material produces a relevant number of ‘secondaries’: other ions, neutrons, electrons, photons, not present in the primary radiation that the shield was trying to stop. Furthermore, this secondary radiation is less energetic and may release more energy (than the original fast radiation) in the matter it travels through. In sum a very careful analysis must be carried on making a passive shield to work properly. If this analysis is not well performed the shield may well worsen the situation. One general result, that might be counterintuitive, is that the light, hydrogenated, materials are the best shield, as they produce less fragments. Most used in space now is Polyethylene while also Kevlar proved to have very good radiation shielding properties. It should be stressed that new materials will not be able to show shielding efficiencies much higher than the ones in use, as these are not far from maximum efficiency. Nevertheless, shielding may be managed with an ‘multi-purpose’ approach, and in this sense new materials that could serve not only as shield, but also, for example, as impact protectors, may be very welcomed.

One important result reached in this field is that most likely only an integrated approach to radiation countermeasures will provide the desired mitigation level, using the vessel shielding, additional passive shielding, possible personal shielding devices, when available active shielding, and all the pharmacological, nutritional and physiological countermeasures.

## The importance of the mission scenario

When studying radiation effects during a deep space mission, the most important parameter to be considered, distinguishing a mission scenario from another, is the duration of the mission.

The astronauts are never exposed to the full power of cosmic radiation. They are either in a vessel, or in a space base. During EVA (Extra Vehicular Activity) the astronauts are protected by a space suit. When they travel, the vessel is exposed to the full radiation, however on ground (Moon, Mars) they will be protected by the celestial body, that will shield about half of the GCR radiation.

Inside a vessel, or a base, the shielding will be provided not only by the external hull, but also by the items positioned inside the habitat, racks, experiments but also all the life support system items such as, for example, toilets. The radiation field inside the habitat will therefore be highly anisotropic. This characteristic can be assumed both for bases and for vessels. The major difference between these two kinds of space habitats is that the former will likely use ‘*in situ*’ materials for shielding, while the vessel may also be built on Earth. Finally, magnetic field (Low Earth Orbit, LEO) and atmosphere (Mars) of the planet must be considered.

In sum, there are physical differences in the habitats that provide different protection scenarios from the radiation perspective:

- Magnetic field
- Shielding by the celestial body where the mission is operating
- Atmosphere
- Additional shieldingAlso, the sources of radiation are varying in time:
- GCR radiation flux is changing, being higher during lower solar activity and viceversa.
- probability of the occurrence of an SPE is somewhat proportional to solar activity.

While developing a mission plan, all the above should be considered. Each of the items above may reduce the radiation effects, in some cases up to about 50%. A full mission will face a mixture of the results coming from the good use of all the characteristics listed above.

As shown by the several available measurements (see below), the order of magnitude of the radiation rate would stay about the same (order of magnitude ≈ 10^-3^ Sv/day).

A reasonable assumption is therefore that the most critical parameter would indeed be the duration of the mission. Trying to exactly determine the details of the mission scenario might be overshooting. It is the length of the mission that must be considered.

## Radiation measurements in relevant sites of the solar system

A collection of Dose Equivalent measurements (Dose Equivalent = Q x Dose, Q quality factor, see Narici et al., 2015, and ICRP, 1990) is provided in **Table 1**: i) in the ISS, ii) during the Mars transit (‘deep space’), iii) on the surface of the moon and iv) on the surface of Mars.

**Table 1:** Radiation measures in several relevant astronomical sites in recent years.

Location	Detector/mission	Year	Ref	Dose Equivalent Rate (mSv/day)	Dose Eq.Rate (mSv/day)estimate (ref 6) solar minimum	Dose Eq.Rate (mSv/day)estimate (ref 6) solar maximum	Note
ISS	Dostel	2016	Berger et al., 2017	0.647	0.73	0.58	
ISS	Dostel	2019	Berger et al., 2020	0.731	0.73	0.59	a
ISS	ALTEA	2012	Narici et al., 2015	0.10 ➔ 0.52 ± 0.01	0.12 ➔ 0.61	0.095 ➔ 0.49	b
Moon surf	LND	2019	Zhang et al., 2020	1.37 ± 0.25	1.4	0.89	
Mars transit	MSL RAD	2012	Zeitlin et al., 2013	1.84 ± 0.33	2.5	1.6	
Mars surf	MSL RAD	2012/13	Hassler et al., 2014	0.64 ± 0.12	0.88	0.57	

a) Unpublished results. This value appears in (Berger et al., 2020).

b) The different values correspond to different directions in the ISS, and therefore to different shielding. Furthermore, ALTEA features a reduced proton and helium ions detection efficiency.

The GCR flux depends to the solar activity [point (v) above], and these measurements describe the radiation at a specific time in the solar cycle. To estimate the possible range of the dose equivalent values in a full cycle, we can use recent data from the variability of radiation due to the modulation by the 11 years long solar cycle (Berger et al., 2020). The available data is the dose, so using these data for extrapolating dose equivalent may introduce an error. However, the modulation of the dose equivalent for protons should follow the one of the dose (Q ≈ 1) while the modulation of Q for LET (Linear Energy Transfer, the energy released in the traversed material measured in keV per traversed µm of materials) ≥ 3 keV/μm is almost absent during the solar cycle (Narici et al., 2015). This suggests that the valuation of the range (at, respectively, the maximum and the minimum of the solar cycle) using the dose data is an acceptable rough estimate.

**Figure 1:**
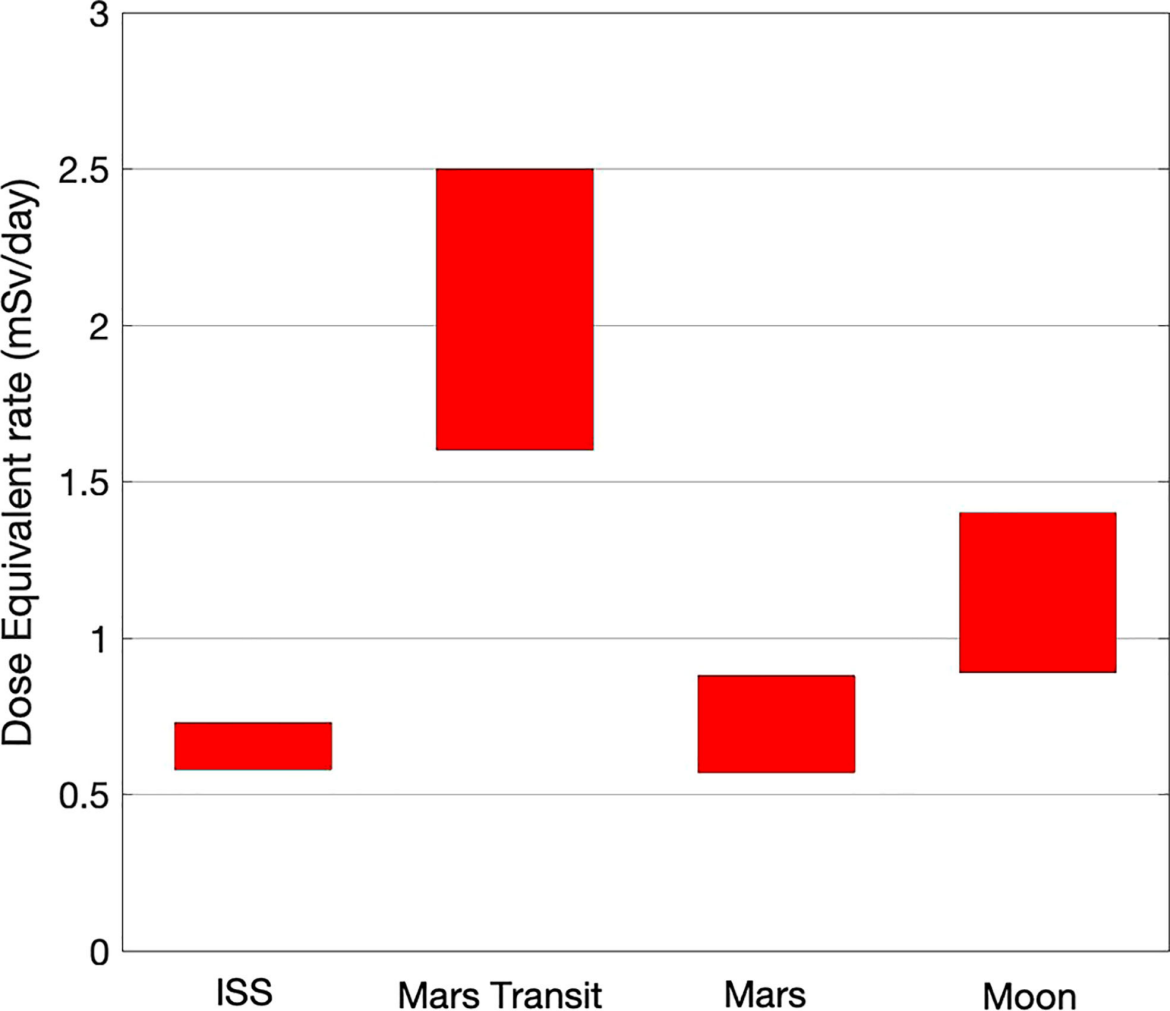
Ranges (maximum-minimum of solar activity) of dose equivalent in relevant sites.

From **Table 1** it is possible to draw the plot in **Figure 1** that provides a rough estimate of the Dose equivalent ranges in the four relevant sites mentioned above.

The Dose Equivalent range extensions in the four sites reflects the different amount of protection: mostly the Earth magnetic field for the ISS, the planet shield plus the Martian thin atmosphere for Mars, just the Moon shield for the Moon, and nothing in deep space.

A few considerations about the plot above should be made.

It should be mentioned here that a factor five of difference in Dose Equivalent has been measured in the high Z component by ALTEA in the ISS (**Table 1**) under different shielding conditions (different directions in the ISS). Even taking into account the contribution of protons to Dose Equivalent, the change in dose equivalent due just to the internal shielding can easily reach a factor 2.

While Dose Equivalent is now a radiation variable widely used, it is also understood that it does not describe the full picture of the health hazards due to space radiation. For this, a more complex risk model will be needed, possibly based also on micro and nano dosimetry, and track structures studies. This leads to observe that a much more detailed assessment of the quality of radiation, which is just roughly described by the Q factors in the Dose Equivalent values, will be needed.

In these possibly strong approximations, the plot in **Figure 1** indicates that ISS is a good analog for the Mars surface, within a factor two of the Moon surface, and within a factor three of the deep space.

The radiation spectra in the four considered locations are similar. The major difference being for ISS the protons trapped in the van Allen belts, making up for about 30% of the dose equivalent (Berger et al., 2017). However, the similarity of the spectra for ions above the protons can be easily appreciated especially if observing the passages of the ISS at the high latitudes, where the Earth magnetic field provides a less efficient protection (Zeitlin et al., 2019).

Radiation fluxes, and consequently dose rates and dose equivalent rates, are quite low. For the mission to Mars (Hassler et al., 2014), it is estimated to be 1 Sv, roughly equally divided among the three parts of the mission: 180 day-trip + 500 day-stay + 180 day-trip. In the average we are therefore at slightly more than 1 mSv/d (see also **Figure 1**).

It is worth noting that the low rate and isotropic nature of the radiation means that it will cover a surface slowly and in a sparce manner. From ISS data (Narici et al., 2017a), when the Station is at high latitude (so the radiation flux is closer to the deep space one) the counting rate on a surface of 1 cm^2^ is about 3 per second.

**Figure 2:**
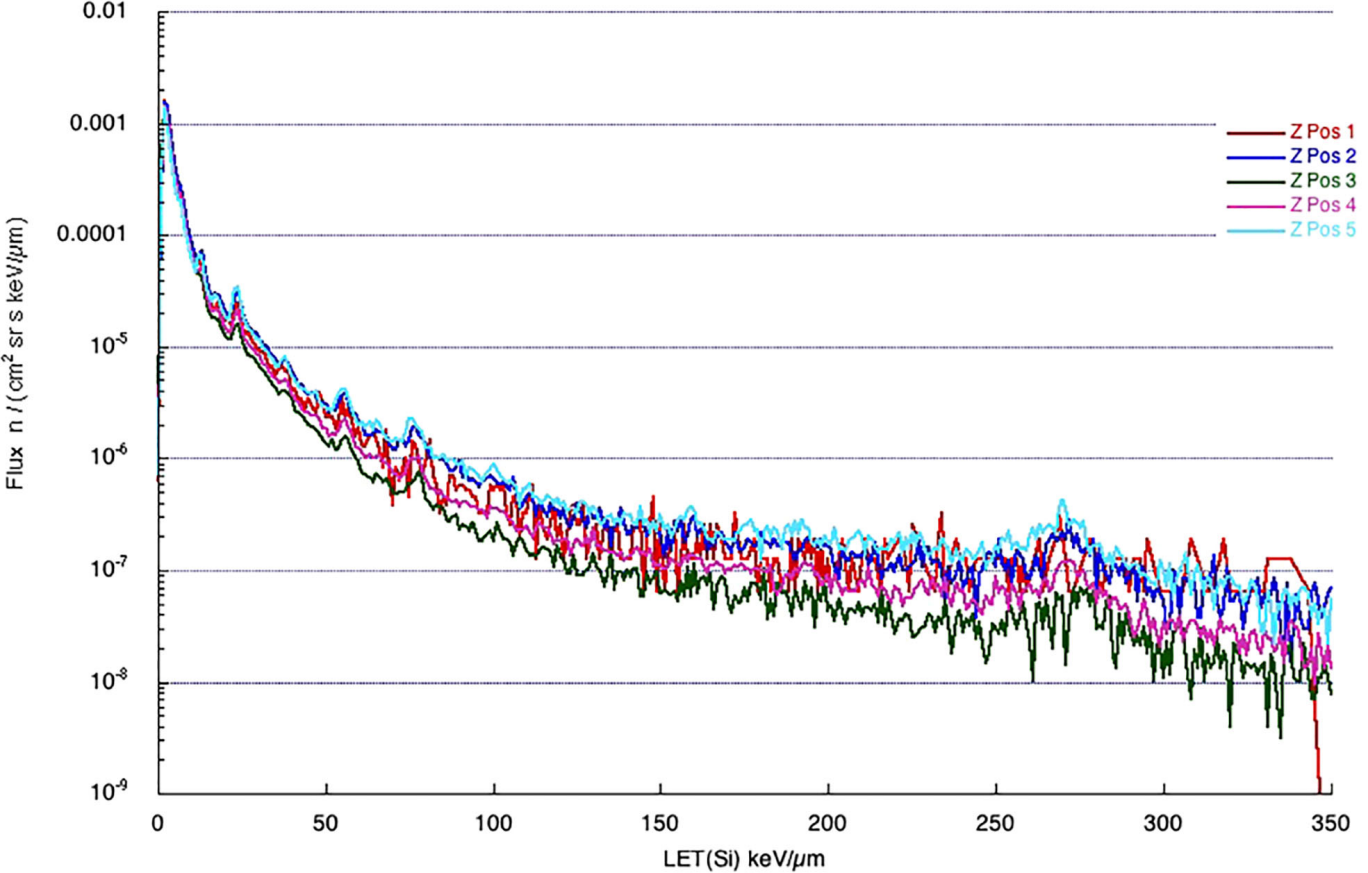
Spectra measured by ALTEA in 2012 in five positions in the ISS (four in the USLab, one, position 5, in Columbus), in the Z direction of the ISS coordinates (from Earth to Zenith). [Reproduced from Narici et al., 2015, https://doi.org/10.1051/swsc/2015037.

**Figure 3:**
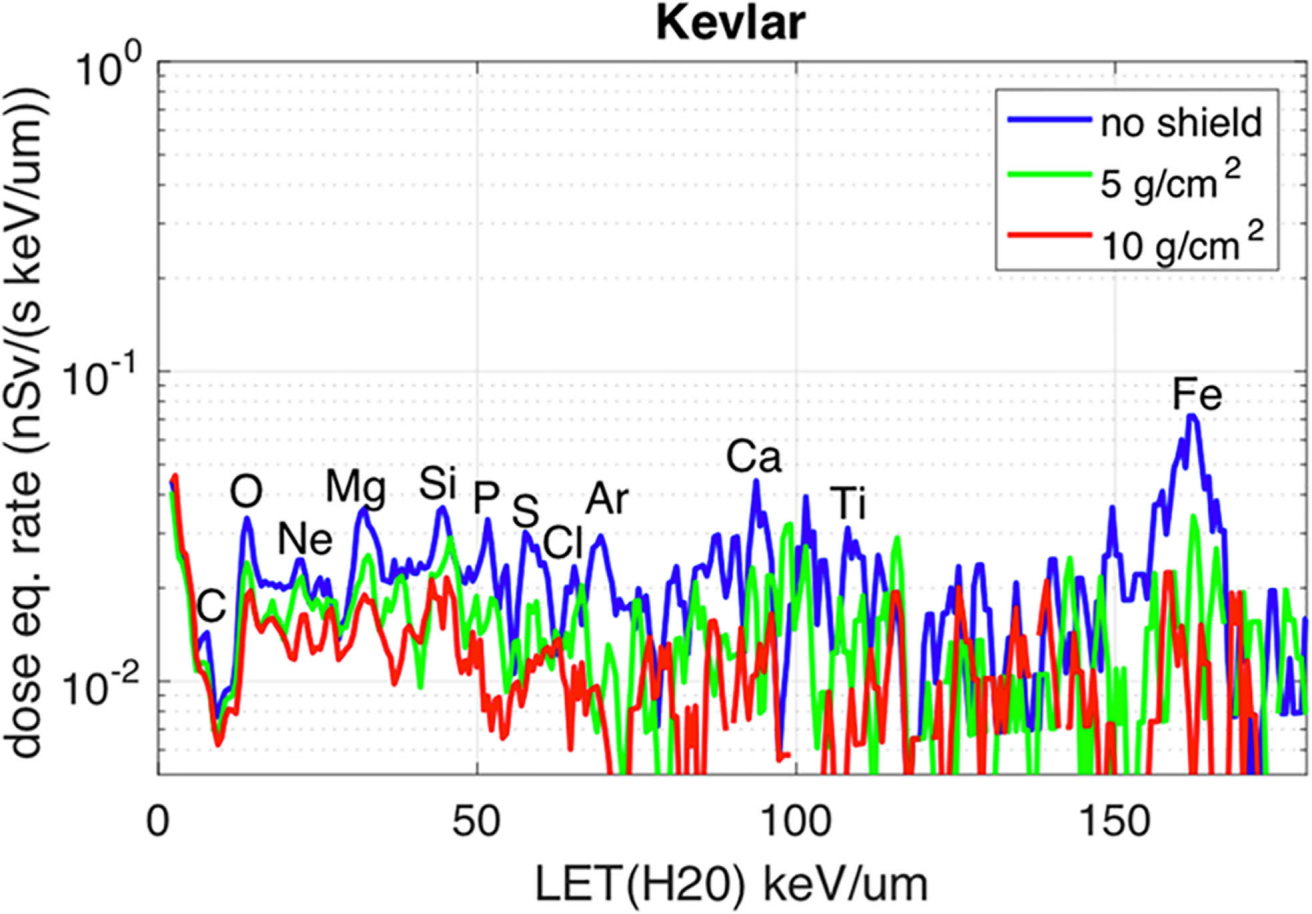
Dose Equivalent measured by ALTEA in the Columbus modulus (position 5 in **Figure 2**), Z direction. Please refer to the blue curve for what is concerned here. The other two curves are related to measurements performed with additional shielding. Most prominent elemental peaks are indicated. [Reproduced from Narici et al., 2017, https://www.nature.com/articles/s41598-017-01707-2].

One of the most important variables linked to the radiation biological effects is the LET. A LET spectra describe the amount of radiation delivering a certain amount of energy to some material, usually silicon (as most of the detectors are made of silicon), per unit length. The LET in silicon is often converted to water to calculate the dose (water has approximately the same density as the human body). The spectra in **Figures 2**, **3** are measured by ALTEA in the 2010-2012 period in 5 different sites in the ISS (Pos 1 to 4 in the USLab, Pos 5 in Columbus). They also suggest the large differences among differently shielded locations. The spectra are about radiation in the Z direction (Earth to Zenith). Note that ALTEA measures only radiation delivering more than 3 keV/µm in silicon (it does not measure most of the protons which deliver less than this threshold). The above position 5 spectrum (in Columbus) (**Figure 2**) can be seen also for the Equivalent dose (**Figure 3**), where the contribution of higher Z ions becomes more prominent due to their Q values (describing the average effectiveness in inducing damages). The spectra in **Figures 2**, **3** show also the effect of additional shielding (in this case 5 g/cm^2^ or 10 g/cm^2^ of Kevlar) on dose equivalent (about -30% and -55% over the full spectrum, respectively) (Narici et al., 2017b).

## Irradiation facilities

To study space radiation effects on cells/molecules/tissues, scientists use ‘space radiation analogues’. Namely they use particle accelerators to irradiate their samples with a known quantity of ‘space relevant’ radiation, studying the effects of the irradiation.

Unfortunately, the ‘space fidelity’ of the ‘radiation analogs’ accelerators is poor. For an accelerator the following issues should be considered:

1. The dose rate is generally many orders of magnitude higher than space relevant.
2. The radiation is more spatially focused than space relevant (again orders of magnitude).
3. The radiation is with single Z, single energy, while space radiation is a mixture of many ions, with a continuum of energies.
4. The radiation comes from a single direction while GCR are isotropic. In addition, another issue, originating from the specific experimental strategy, should be mentioned:
5. The dose required to measure the effect under study may be much higher than space relevant.

The above issues should be individually addressed asking what the relevance of the specific ‘non-fidelity’ in the biological model under study is.

As the biological relevance of the above issues is not well understood, the non-fidelity of the ‘accelerator analogue’ certainly produces difficult-to-quantify uncertainties in the final results. These uncertainties are likely larger than those produced by the possible different mission scenarios, once the mission duration is determined. This supports the idea that to assess radiation risks the needed parameter to describe the mission is its duration.

The above issues are approached in several ways.

The first (1) is well recognized: dose rate in accelerator experiments is about 6 order of magnitude higher than in space (roughly 2 years of exposure to space radiation is often compressed in 1 min). A very rough figure for “space relevant” could be 10^-1^ Gy delivered in 2 y. The complete solution of this problem is probably unfeasible as it brings in a number of experimental issues. To fully mimic space radiation rate, the experiment should be approximately of a similar duration of the space mission it tries to mimic, and this is obviously not feasible for many reasons. Nevertheless, the 6 order of magnitude difference could be reduced to 2 order of magnitude with experiments lasting one week, or to a single order of magnitude, with experiments lasting 2.5 months. And, maybe, these could be more feasible for some experimental approaches. There is no real technical limit to lower the accelerator rate, however the limits are in keeping a biological experiment running for this long as well as cost reasons. Nowadays, especially at the Brookhaven NSRL facility, fractionated experiments are tried as a mitigation strategy for these issues. Plans to mitigate the effects due to this issue are under study in a other few major facilities. However, it is important that the experimenters consider this possible cause of errors when planning and analyzing experiments.

The second issue (2), linked with the first one, is possibly not relevant for radiobiology experiments, nevertheless, it is totally overlooked in planning experiments. Experimenters should be aware that in space we may have in one second ≈ 3 ions/cm^2^. Under irradiation there might be 10^8^ ions/cm^2^, decreasing the average distance of two hits in one second from cm to µm. The relevance of this issue should be recognized, checked and stated.

Both these first two issues deal with the required beam-time to carry on the experiments. This is a parameter that the scientists may try to minimize for reasons going from mere costs to logistic feasibility to the actual experimental feasibility. Shortening the beam time, however, increase the impact of the two issues discussed above

The issue (3) is the one that is having the strongest worldwide answer: GCR (and SPE) simulators are being developed in several accelerators. (Norbury et al., 2016). Several energies of each ion and more than one ion in rapid succession (few minutes) can be delivered at the GCR simulator of the NSRL-BNL (USA) (Norbury et al., 2016). At GSI (Darmstadt, Germany) a different approach for a GCR simulator is followed and will use pre-settled fragmentation to deliver a radiation spectra similar to the space one.

The issue (4) is not recognized, but might have a moderate impact on radiobiology experiment, while the issue (5) is strictly linked to the way an experiment is designed. As a general rule either an extrapolation to low doses – low dose rates of the results should be planned (and therefore feasible), or, whenever possible, the experiment should be re-designed to allow for more space relevant radiation parameters.

All issues and possible solutions should be approached for each specific experiment, to understand if each absence of fidelity proper of the irradiation system may impact the results of the investigation:

- Distance in time between two successive ions
- Geometrical distance between two successive hits
- Possible non linearities of the effects due to different ions (Z) or/and energies
- Directions of the impinging radiation

## The evolution of the interest in the studies on radiation effects on plants

The interest in studying the effect of Space factors, especially radiation, on plants has increased in the last decades because of the crucial role of plants as regenerators of resources in BLSS in long-term manned missions. In such hybrid closed systems, based on the integration of biological/physical/chemical subsystems, higher plants can contribute to air regeneration by CO2 uptake and O2 production (through photosynthesis), water purification (through transpiration), recycling of wastes of the crew, and food production (in case the plants cultivated are edible crops) (Wheeler, 2003; De Pascale et al., 2021). The need for plant cultivation in Space is not only related to material needs but also to healthy issues. Indeed, plant-based fresh food produced in Space can represent a good strategy to support the astronauts’ diet, providing food with high nutritional value and rich in antioxidants. Moreover, it is recognized that the conditions of isolation suffered from astronauts are mitigated by the presence of plants in the pressurized modules of space platforms, especially during the activities of Space farming (De Pascale et al., 2021). Indeed, the introduction of plant-based fresh food in astronauts’ diet is promising to improve crew health in so far as nutrition is considered a fundamental countermeasure to counteract Space-induced diseases, including bone and muscle mass loss, anorexia, appetite change and alteration of feeding behavior, and other metabolic stresses (Bergouignan et al., 2016). Moreover, another hypothesis is emerging that the exposure of plants to radiation during cultivation may improve the nutraceutical properties, especially the antioxidant value, of edible organs (De Micco et al., 2021a; De Micco et al., 2021b). Therefore, the exposure to space ionizing radiation during the activities of Space farming, within certain limits, would become a sort of “Space cultivation factor” for crops stimulating the production of fresh food enriched in antioxidant content which assumed by astronauts with diet may represent a further countermeasure to improve their physiological defenses. However, it is necessary to achieve fundamental knowledge on plant physiological behavior within the radiation environment the crops will encounter.

Available knowledge on plants’ response to radiation suffers from the difficulty in comparing data deriving from experiments using different models, conditions and parameters (related to both the plant material and the radiation source). Up to now, most of the research has been conducted in the medical field on animal models, where ionizing radiation has been increasingly used for both diagnostic and therapeutic purposes (Tinganelli and Durante, 2020). Regarding plants, studies available in literature were often designed with different purposes and applications, ranging from topics related to the breeding industry up to radioecological issues at NORM (naturally occurring radioactive materials) sites or consequent to nuclear accidents (De Micco et al., 2011; Ludovici et al., 2020; Mrdakovic Popic and Skipperud, 2020). The effects of ionizing radiation on plants have also been studied because radiation is considered a tool to select cultivars with improved crop yield and quality in breeding programs and is a decontamination mean to sanitize seeds and food. In the last decades, the ecological accident of Chernobyl in 1986 and the more recent disaster of Fukushima in 2011, have raised more and more attention towards radioecology: the branch of ecology which studies the presence of radioactivity in the ecosystem. Among the others, radioecology focuses its attention on the mechanisms by which non-human organisms counteract the effects of ionizing radiation (Arena et al., 2014a). Furthermore, the relationships between low doses of radiation, early effects and risks for plants and animals are also investigated in a sort of risk assessment to understand the impact of radiation on the environment (Brown et al., 2008).

**Figure 4:**
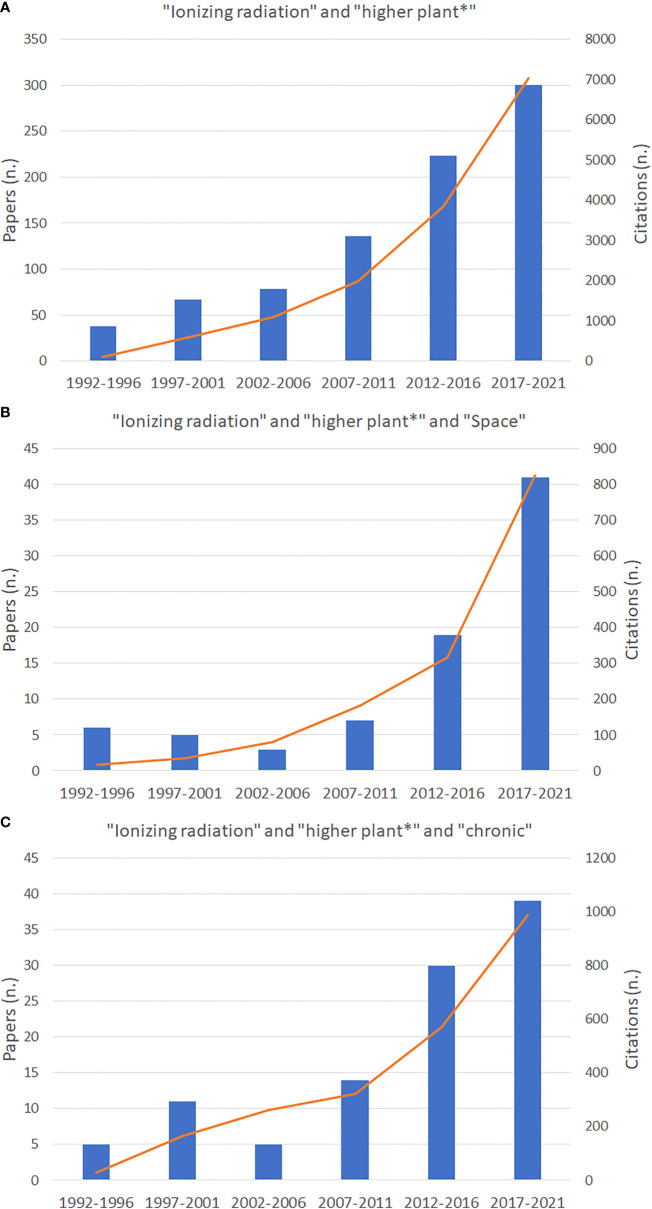
Number of papers published from 1991 to 2021 and related citations dealing with ionizing radiation and higher plants **(A)**, and Space **(B)**, and chronic radiation **(C)**, as indexed in the Web of ScienceTM database (Thomson Reuters).

Nowadays there is increasing awareness on the need for gaining data from specific Space-oriented experiments taking into account the different mission scenarios. Papers dealing with the effect of ionizing radiation on higher plants have progressively increased in the last 30 years as shown by data on the number of published papers and citations from Web of ScienceTM database (Thomson Reuters) in the period from 1992 to 2021 (**Figure 4**). Graphs in **Figure 4** indicate that in the last five years, compared to the previous quinquennium, the increase in papers dealing with “ionizing radiation” and “higher plants” in general has increased only by 26%, while the increment of all papers dealing with “ionizing radiation” and “higher plants” in “Space” has increased by 54%. This suggests a phenomenon by which more knowledge on plants’ response to radiation is produced in Space-related science compared to other research fields. It is also evident that the papers dealing with “ionizing radiation” and “higher plants” in “Space” represents only 13% of all papers published on higher plants’ responses to radiation. As well, papers dealing with chronic radiation account for 14% of all published papers on “ionizing radiation” and “higher plants”. The lack of papers on chronic effects depends on two main phenomena, namely the reduced availability of irradiation facilities for chronic irradiation for ground-based research, and that experiments conducted in Space have often been reported focusing on the effects of microgravity on very specific plant growth processes, without considering that such responses derive instead from the interaction between microgravity and other environmental factors including chronic exposure to ionizing radiation, that cannot be completely shielded in Space platforms, and to secondary radiation deriving from the interaction between cosmic radiation and facilities themselves.

The rising interest in understanding the effects of ionizing radiation also on organisms other than mammal models is dictated by the long-term Space exploration objectives of the main international Space Agencies. Indeed, among the exploration scenarios objectives of Global Exploration Roadmap (GER), issued by the International Space Exploration Coordination Group (ISECG), the development of infrastructures to ensure long-duration habitation is listed (ISECG, 2020). Therefore, understanding the effect of ionizing radiation on plant growth represents a critical issue to evaluate the impact on the functioning of Bioregenerative Life Support Systems (BLSSs) that will represent the most suitable solution for human settlements in extraterrestrial environments (Yu et al., 2007; De Micco et al., 2011; Arena et al., 2014a).

## Target and endpoints in the short- and long-term missions

In the past decades, the studies on the effects of ionizing radiation on higher plants have been focused on several endpoints especially regarding genetic alterations (including chromosomal aberrations, formation of micronuclei and mechanisms for repair) and recent technological development has allowed highlighting more and more details of possible molecular and chromosomal damages compared to the first studies (De Micco et al., 2011; Ludovici et al., 2020). At morpho-functional level, investigations have been addressed to endpoints in growth and reproduction processes by analyzing germination, mortality, lethality, organ morphogenesis, photosynthesis, biomass partitioning, flowering and production of seeds for the next generation. It has been recently claimed that in the effort of understanding plants’ responses to radiation, we must not lose sight of the main differences between animal (for which much more information is available) and plant models which likely explain the higher radioresistance of plants compared to mammals (Arena et al., 2014a; Arena et al., 2017). Such differences can be ascribed to two main points. The first regards plant metabolism in which many processes, such as light reactions of photosynthesis normally produce reactive oxygen species (ROS) that plants are accustomed to block due to the large production of antioxidants. The second regards the growth mechanisms relying on meristems, reproductive processes based on the alternation of sporophytic and gametophytic generations, as well as the lack of a circulatory system which altogether make plants not suffering from the same stochastic and carcinogenic effects as animals (Ludovici et al., 2020). However, it has been recently highlighted that although stochastic effects arising from chronic low doses of ionizing radiation may be of little relevance in non-human biota, the issue is still under debate and stochastic effects in plants may be variable between individuals but also at intra-individual level due to different radiosensitivity of different organs (Esnault et al., 2010; Caplin and Willey, 2018). Furthermore polyploidy, common in higher plants, is considered a property inducing higher resistance to organisms because it is unlikely that radiation particles hit the very same sequence of DNA and the existence of additional copies of the genome can hide recessive mutations (Bhaskaran and Swaminathan, 1960; Gartenbach et al., 1996; De Micco et al., 2011). The lower radiosensitivity of polyploids suggested in ground-based experiments would be likely even more relevant in the case of the low-rate, isotropic space radiation. Low rate of Space radiation would allow to DNA repair mechanisms to restore the early DNA damages before another damage occurs.

### Are all plant tissues really resistant to radiation?

The supposed, and partly demonstrated but also probably biased, belief that plants are very resistant to radiation might be due to the fact that most experiments on plants have been conducted having dry seeds as target for irradiation, using acute doses due to logistic limitations mainly linked to available volume in irradiation facilities and limited beamtime (Shi et al., 2010; De Micco et al., 2014a; Arena et al., 2017; Arena et al., 2019). However, the “dry seed stage” is indeed a peculiar status of the plant life cycle, characterized by the highest resistance to environmental stressors, due to the seed structural and metabolic traits (De Micco et al., 2011). During the evolutionary history of higher plants, the dry seed status has been “designed” to resist to the most constraining environmental conditions to guarantee the species survival. The high radio-resistance of dry seeds can be however rapidly lost during germination. A major part of the radio-resistance of dry seeds to radiation can be ascribed to their peculiar state of dehydration in so far as the water deprivation is known to limit the radiolysis and, in turn, the overproduction of free radicals (Hase et al., 1999; Kim et al., 2011). The overproduction of free radicals is considered among the first steps in the process of injury arising in biological molecules/cells/tissues/organs. In fact, the water molecule, highly represented in all organisms, is a main target of both direct (radiation energy deposited into the target) and indirect interactions (radiation energy pass through a medium being scattered in diffusible intermediates) between radiation and biological targets (Esnault et al., 2010). Water ionization itself and direct interactions also on other biological molecules (including DNA) activate the cascade process responsible for the production of ROS scavenger enzymes (Esnault et al., 2010 and references therein). Therefore, the high radio-resistance of dry seeds is rapidly lost during germination because of the loss of the dehydration protection. Moreover, the process of seedling survival and establishment, both under field natural conditions and in controlled cultivation, is one of the most critical and vulnerable phases in the plant life cycle, and it can be seriously affected by both intrinsic and environmental factors (De Micco et al., 2014b). Until seedlings reach photo-autotrophy, post-germinative development is based on the storage of reserves, such as carbohydrates (usually starch), protein and lipids. These compounds are mobilized into soluble metabolites, allowing growth and establishment before seed nutrients are completely depleted (Gommers and Monte, 2018). The direct interaction of radiation with such molecules as well as the overproduction of reactive oxygen species (ROS) can interfere with structural and functional organic molecules, including proteins, lipids, starch and nucleic acids, causing disturbance to the cellular metabolism, which in turn may compromise the efficiency of reserve mobilization and finally the seedling survival (Ahuja et al., 2014). Another trait suggesting the higher vulnerability of germinating seeds is the large occurrence of actively growing tissues where faults in the cell cycle can be quickly multiplied with consequences on organogenesis (De Micco et al., 2021b). Different responses in the same species have been reported when the target organ is at different stages of development (actively growing or adult leaves) at the time of exposure (Arena et al., 2014b; De Micco et al., 2014c). Similarly, to meristematic tissues, also cells involved in the production of megaspores and microspores can be important targets to be protected by radiation injury, and possible radiation-induced mutations, because the success of mega- and micro-sporogenesis is one of the needed steps to achieve the completion of the ontogenetic cycle culminating in seed production in flowering plants, thus supporting the perpetuation of the species in the next generation. Indeed, the production of viable seeds is a target to be pursued in the long-term missions to ensure the crew to obtain enough seeds to continue cultivation.

### Main critical areas for plant-based BLSS design and related scientific plant biology issues

Understanding how space radiation impacts specific plant growth processes is important not only for the advancement of fundamental knowledge of plants’ responses to radiation, but also for the impact on BLSS design. The main endpoints to be studied are still numerous before achieving an overall understanding of plants’ response to radiation, but some priorities should be identified. In the short term, the main endpoints to be analyzed are those applied to the air regeneration in BLSS (i.e. photosynthesis for carbon fixation and oxygen generation) and food production (i.e. plant growth and organogenesis for biomass production) as well as nutritional traits of produced edible biomass.

#### Ionizing radiation and photosynthesis

Photosynthesis is a complex process that may be altered at any step by ionizing radiation: electron transport carriers (i.e. photosystems and cytochromes), light-harvesting pigment-protein complexes and enzymes of the carbon reduction cycle (Hou et al., 2017). The photosystem II (PSII) is one of the main targets of radiation because the core of PSII, constituted by D1 and D2 proteins is very sensitive to injuries. More specifically any damages to D1 protein slows down or inhibits electron transfer between the primary electron donor and the secondary plastoquinone acceptor. The degree of PSII damage is influenced by light environment, being more pronounced at high light conditions which favor the photoinhibition of the photosynthetic apparatus (Giardi et al., 1997; Esposito et al., 2006). However, the excess of light would not be a problem in BLSS if levels of light intensity are kept below the species-specific photo-inhibition levels due to energy budget constraints. The interaction between light intensity and radiation should be instead considered when adopting strategies to maximize crop productivity by increasing photosynthetic photon flux (Bugbee and Salisbury, 1988). All the components of the photosynthetic electron chain are vulnerable since the exposure to both acute and chronic doses of X and gamma radiation induces oxidative damages due to an over-production of reactive oxygen species (ROS) (Zaka et al., 2002). The light harvesting complexes may be also compromised following a dilation between thylakoid membranes and the occurrence of defective chloroplasts (Cheng and Chandlle, 1999; Abe et al., 2002). As a consequence of chloroplast alteration, chlorophyll depletion may occur together with a reduced capability of light harvest by the whole antenna system (Palamine et al., 2005).

Several studies demonstrated a close inversely proportional relationship between gamma rays dose increase and photosynthetic pigment content (chlorophylls and carotenoids) in maize and lettuce plants sprouted by dry irradiated seeds (Marcu et al., 2013a). However, there is also evidence for radiation-induced either increase or decrease in the levels of photosynthetic pigments. Seedlings developed from irradiated corn seeds increased the synthesis of chlorophyll and carotene (Vlasyuk and Marina, 1970). Differently, Fan et al. (2003) in alfalfa sprouts did not observe any differences in photosynthetic pigments. Generally, gamma doses from 2-70 Gy enhanced the photosynthetic pigments in plants sprouted by dry seeds while higher doses reduced the pigment content (Marcu et al., 2013b). Carotenoids plays an important role as detoxicant agents in radiation damage and ROS scavenging (Fukuzawa et al., 1998). An age and dose dependent increase was observed in carotenoids content in *Cullen corylifolium* leaves being maximum at pre-flowering followed by flowering and post- flowering stages (Jan et al., 2013), even if other studies showed a strong variation of these trends among cultivars of *Capsicum annuum* L. (Kim et al., 2004).

It has been also demonstrated that the expression and the activity of Rubisco was seriously impaired by high doses of X-rays in dwarf bean; these damages were more evident in young compared to adult leaves (Arena et al., 2014b). Indeed, since young leaves receive irradiation while actively growing, possible injuries may be quickly propagated through cell cycles leading to more severe response compared to adult leaves. This would explain the severe dose-dependent changes in morphological and biochemical traits in young leaves and the implementation of a strategy to cope with radiation, based on the enhancement of both DNA repair and antioxidant content with radical scavenging activity (Arena et al., 2014b). It is noteworthy that the ability to develop protection mechanisms and the capability of repairing damage at photosynthetic apparatus level, contributed significantly to the radioresistance mechanisms of a given species (Caplin and Willey, 2018). The past and current studies provide evidence that ionizing radiation may modify plant capability to harvest light, affecting at different extent the pigment-protein antenna complexes. This leads to an important consideration: species which are able to maintain the stability of photosynthetic pigment pool have good chances to overcome the injuries of radiation replacing the functionality of photosynthetic apparatus.

#### Ionizing radiation and antioxidant charge in plants: consequence on nutritional value

One of the main reasons for the higher resistance of plants to radiation compared to animals is due to the many protective mechanism plants have evolved to counteract the oxidative stress (Arena et al., 2014a). Higher plants are able to modulate different metabolic pathways, often resulting in the overproduction of antioxidant compounds, including phenolics that are synthetized in the phenylpropanoid pathway. This route can be considered one of the most important for the colonization of the terrestrial environment by land plants since it is related to the synthesis to many organic compounds useful to help plants in overcoming the new constraints of a life not protected by water. Among these constraints, high solar radiation was the most critical because the past atmosphere was different from the current and phenolics may likely have had a fundamental role in filtering the UV-B radiation avoiding irreversible damages on plant tissues (Graham, 1993). It has been suggested that in an imaginary vision, higher plants in Space exploration could again face environmental conditions similar to remote past times when radiation levels were much higher (Graham, 1993; De Micco et al., 2009). The high responsiveness of antioxidant production in plants has an evolutionary basis. Indeed, during evolution, in the conquest of land, plants had to face multiple environmental constraints including variability in water availability and increased exposure to radiation, priming dramatic modifications in cellular, physiological, and regulatory processes. The development of metabolic pathways to produce antioxidants, and other compounds like protective proteins, is associated to the avoidance of photo-oxidative damage and neutralization of free radicals (Rensing et al., 2008). The increase in antioxidant compounds is therefore a consolidated strategy to help the plant withstanding a number of environmental stresses (e.g. drought, excess light, extreme temperatures, pollution, nutrient deficiency, and radiation itself) by reducing the oxidative stress through removing ROS (Mehla et al., 2017). Indeed, among defense strategies against radiation, the antioxidant pool is very active in the developing of radioresistance in plants. The exposure to ionizing radiation significantly influences the enzymatic oxidative defense systems inducing the activity of glutathione reductase and peroxidase (GR and GPx), superoxide dismutase (SOD) and catalase (CAT). Even the non-enzymatic antioxidants are involved in cell protection against the ROS injuries. It has been observed that the slight increase of scavenger enzymes activity, after a radiation exposure event in some plant species is a regulation mechanism to reduce ROS concentration below dangerous levels, but high enough to activate detoxification defense pathways (Arena et al., 2014a; Vitale et al., 2021).

The synthesis of naturally occurring compounds, phenolics, ascorbic acid, vitamin E, carotenoids, anthocyanins, glutathione is stimulated by ionizing radiation and this phenomenon may have a considerable potential. Consumption of fresh plant rich in phytochemicals and antioxidants has been reported to overcome some of the degenerative diseases that affect humans. For example, plants of *Solanum lycopersicum* L. ‘Microtom’ from seeds exposed to 25 Gy Ca ions developed larger fruits with higher values of SOD activity and richer in carotenoids, anthocyanins and ascorbic acid than control (Arena et al., 2019). Recent studies also showed that the morpho-physiological response of plants to increasing doses of X-rays delivered to germinated seeds is strongly influenced by the light quality during cultivation. In particular, total flavonoids content was the highest in seedlings developed under a mix of red and blue light after being irradiated at the dose of 20 Gy X-rays compared to 0.3 and 10 Gy (De Micco et al., 2021b). More specifically, given the same photosynthetic photon flux density (PPFD) during seedling development, the mix of red and blue light increased total flavonoids content at all doses compared to monochromatic red and white light, but the increase of flavonoids at 20 Gy was maximum (De Micco et al., 2021b). In the case of irradiation with 25 Gy of Ca heavy ions, a radiation-induced decrease in total flavonoids was found which was less severe when plants were cultivated under red-blue than full-spectrum light with the same PPFD (Vitale et al., 2021).

Generally, the exposure to very low doses of ionizing radiation, can favor the activation of many protective mechanisms, both at organ and cell level, being responsible of positive outcomes. The complexity of these phenomena is referred to as “hormesis” (De Micco et al., 2011; Arena et al., 2019). Examples of hormesis are faster germination, increased phenolic compounds and phytochemicals which represent an added value for vegetables cultivated for food purposes. However, such a phenomenon suggests a pre-acclimation mechanisms that improves the plants’ ability to respond to a second stressor as happens after fractional irradiation or irradiation with chronic exposure (Esnault et al., 2010).

## Acute *vs.* chronic doses

Although not always quantitatively comparable in terms of radiation parameters and protocols, the studies conducted in the last 30 years have helped some outlines to develop (Esnault et al., 2010; De Micco et al., 2011). The severity of radiation damage on plants depends on the species itself and plant developmental and physiological status at the time of irradiation as well as on the radiation type, exposure time (acute or chronic), dose, dose rate and other features of the radiation source. Ionizing radiation with high linear energy transfer (LET), such as protons and heavy ions, is more harmful to plants and animals compared to low-LET radiation (X- and γ-rays). Concerning the exposure time, most of available information for non-human organisms refers to acute exposure. The dose of exposure can induce very different responses in plant and mammalian cells: the same dose can be considered “high” and induces detrimental effects in mammals but not in plants (Arena et al., 2014a). In human tissues, the 0.1 Gy dose, for both high- and low-LET (Linea Energy Transfer) radiation, has been recognized as a threshold dose for deterministic effects (ICRP, 2007; Arena et al., 2014a). The same dose range is considered very low for the majority of plants, often showing no detrimental effects. Just to mention a few studies, De Micco et al. (2014a) found a high radioresistance in dwarf tomato (*Solanum lycopersicum* L. cv. ‘microtom’), with no alteration on morpho-anatomical and photosynthesis from 0.3 up to 100 Gy X-rays, delivered at the stage of dry seeds. Similar results were found in *Phaseolus vulgaris* L. adult plants subjected to the same X-ray doses, which did not show any perturbations in leaf structure even at the highest level of irradiation (De Micco et al., 2014c). Data from experiments using acute doses of irradiation show often contrasting results with findings from the fewer experiments concerning low-dose-chronic-exposure (Coppelstone et al., 2008; Mousseau and Møller, 2020), often replaced by estimations based on RBE (relative biological effectiveness) modelling (Alonso et al., 2008 and references therein; Caplin and Willey, 2018). However, the Chernobyl and Fukushima areas after the nuclear accidents as well as area surrounding the nuclear test site in Semipalatinsk, Kazakhstan, have represented and still represent natural open-field laboratories where to study the effects of chronic radiation on several wild and crop species. Studying the effects on plant populations at different distances from the nuclear power plants and in different years have represented a way to evaluate the short-term effects of high acute doses (corresponding to closer areas in the periods immediately after the accidents), low acute doses (in areas at increasing distance in the periods immediately after the accidents) and long-term effects of chronic irradiations in the years subsequent to the accidents (Esnault et al., 2010; Ludovici et al., 2020). Some of the trends identified suggest that radiosensitivity declines with age, as well as chronic low dose irradiation is more harmful per unit dose than high-dose irradiation (Caplin and Willey, 2018; Ludovici et al., 2020). For instance, Zaka et al. (2002) found that the chronic exposure of Stipa capillata seeds to γ and β radiations led to a higher expression of scavenging enzymes, DNA-repair genes and antioxidants in plant tissues. Whereas, field studies conducted in the Chernobyl area, showed that the accumulation of γ radiation through time, provoked unrepaired damage in pines, such as reduced growth and morphological alterations in needles (Sparrow et al., 1970; Real et al., 2004; De Micco et al., 2011). Another emerging issue is that radioresistance, adaptive response and evolution can arise when plants are submitted to systematic or repeated irradiations events (Esnault et al., 2010). Indeed, a first stress induced by ionizing radiation has been shown to increase the plants’ ability to cope with a secondary stress either caused by radiation itself or due to other stressors (Zaka et al., 2002; Baek et al., 2005). Two hypotheses have been formulated to explain such radiation-induced increased resistance to stressors, the first linked to the signaling operated by secondary ROS (mainly H2O2), the second involving secondary metabolites (Esnault et al., 2010).

The different responses to acute and chronic irradiation may also be due to a different genome regulation as transcriptome analysis have revealed that different groups of genes are activated/inhibited in the two types of radiation (Kovalchuk et al., 2007).

Caplin and Willey (2018) have recently made the effort of reviewing the issue of radioprotection in plants referring to the set of reference animals and plants (RAPs) developed by the International Commission on Radiological Protection (International Commission on Radiological Protection, ICRP, 2008) and discussing several acute high- and chronic low-dose data against Derived Consideration Reference Levels (DCRLs) for each RAP (International Commission on Radiological Protection, ICRP, 2014). They also clearly reported that more research is needed on radiation effects on plants to broaden present knowledge on a wider range of plant species.

## Conclusion and future approaches

A sustainable human presence in deep space requires BLSS. However, the presence of high levels of ionizing radiation in extraterrestrial environments impacts the biology of chosen crop species, as well as the materials and electronics. Therefore, since one of the main challenges is ensuring the productivity of edible plants in a BLSS in these environments, there is an urgent need to increase available knowledge on the responses of higher plants to radiation.

In the design of unmanned space greenhouse modules, envisaged for vehicles, orbital or planetary platforms, the shielding needs of greenhouses must be based on the knowledge of plants’ response to acute and chronic doses of ionizing radiation. Although data available in literature are not easily comparable, two phenomena are clear: 1) plants are more resistant than mammals, and 2) radiation, especially when combined with other specific levels of environmental factors (e.g., light quality), can induce protection mechanisms at specific life stages which can also improve the nutritional value of edible parts. Knowing in-depth plants’ tissue-specific radio-resistance in the various mission scenarios is necessary information that may allow a reduction of shielding requirements (with consequent reduced mass and economic constraints) in case of automatized greenhouses.

Considering that available knowledge derives from the analysis of data mainly obtained with X- and Gamma-rays and/or with charged particles different from and doses higher than those found in the space environment, the first step to boost the research on plant radiation effects is to validate available knowledge with Space-relevant radiation in the different mission scenarios. This would allow solving the issues of properly defining the shielding needs, the cultivation requirements and protocols, as well as solving the incongruity still present in literature.

Open issues in plant radio-resistance are still numerous and regard fundamental science and applied objectives including:

- understanding the basic mechanisms for radio-resistance in different plant forms and species. This is fundamental to guide crop management starting from the choice of species and cultivars up to the modulation of the cultivation factors. Indeed, the proper management of cultivation factors would help improve the plants’ tolerance to abiotic stresses to achieve a sort of RAD-HARD plant suitable for cultivation in Space outposts.
- evaluating how plant behavior and productivity (including the ratio between edible/not edible biomass) can be altered due to radiation thus changing its capability to act as “resources regenerator”. Such knowledge is fundamental in order to define the requirements for plant cultivation in space greenhouses (for examples, refer to Bamsey et al., 2009; THESEUS roadmap, 2012; ESA SciSpacE White Papers, 2021).
- considering the possibility to produce fresh food onboard characterized by radiation-induced increased content of anti-oxidant compounds to improve physiological defenses of astronauts. This is a sort of production of functional food after the exposure to ionizing radiation and in such a view, radiation is transformed from a constraint into an opportunity.

**Figure 5:**
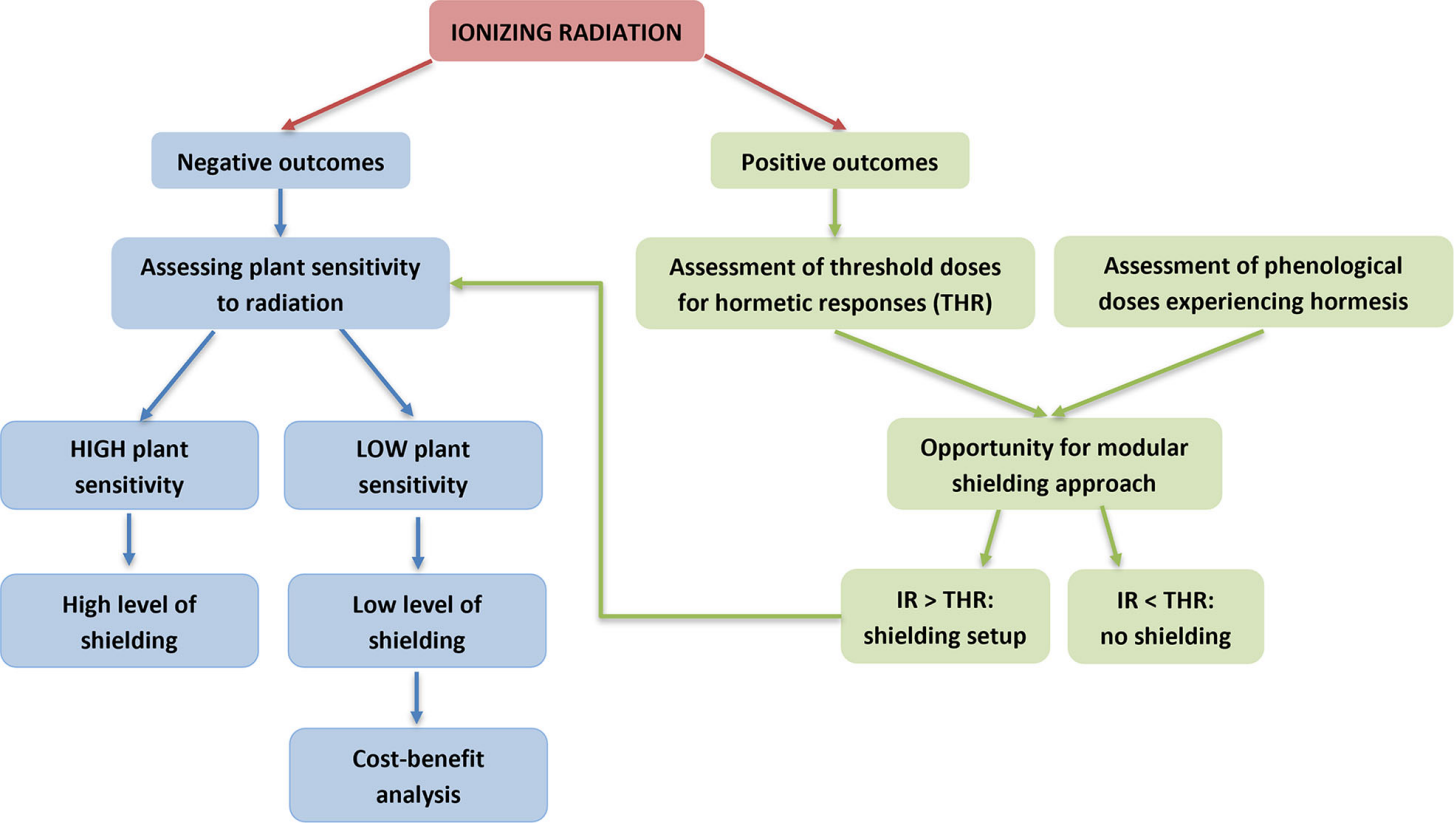
A flow chart for definition of shielding requirements for plant growth in Space in relation to sensitivity to ionizing radiation.

Considering that the road is still long to cover and that resources invested are limited, to optimize scientific efforts, an idea is to follow a systematic approach as a chart to define the shielding requirements for plants in each key developmental stage (**Figure 5**). The first question to answer is whether the effect of ionizing radiation is negative or positive. A negative effect triggers the second question, namely what is the degree of sensitivity of the plants. In such a case, high sensitivity means the need for high level of shielding, while low sensitivity means the need for low level of shielding. Low sensitivity can even open the way towards a benefit-cost analysis on shielding strengths (improvement of quantity and quality of yields) and weaknesses (costs and technical constraints). In the case positive effects occur, the threshold dose at which the hormetic effect happens (THR, threshold doses for hormetic response) as well as the phenological stages at which hormesis occurs must be evaluated. At those phenological stages experiencing hormesis, irradiation below the THR can be translated into no need for specific shielding requirements. In case irradiation overcomes THR, plant sensitivity to radiation must be assessed to define the level of shielding. Such a systematic decision-making flow opens the way towards an approach for shielding which might be modulated depending on variable plants’ needs.

## Author contributions

VDM and LN conceived and designed the manuscript. VDM and LN wrote the main part of the manuscript. All authors wrote specific parts of the manuscript, revised and approved the submitted version of the manuscript.

## Funding

This research was supported by the project “*In situ* REsource Bio-Utilization for life Support system (ReBUS)”, unique project code (CUP) F74I16000000005 funded by the Italian Space Agency (ASI), (DC-VUM-2017-080 “Bando di Ricerca per missioni future di esplorazione, umana dello spazio - Area tematica “Sistemi Biorigenerativi”).

## Acknowledgments

This research benefited from fruitful discussion within the project “*In situ* REsource Bio-Utilization for life Support system (ReBUS)”, funded by ASI.

## Conflict of interest

The authors declare that the research was conducted in the absence of any commercial or financial relationships that could be construed as a potential conflict of interest.

## Publisher’s note

All claims expressed in this article are solely those of the authors and do not necessarily represent those of their affiliated organizations, or those of the publisher, the editors and the reviewers. Any product that may be evaluated in this article, or claim that may be made by its manufacturer, is not guaranteed or endorsed by the publisher.
